# Early Peritonitis Within the First Six Months and One-Year Mortality in Elderly Peritoneal Dialysis Patients: A Multicentre Retrospective Study

**DOI:** 10.3390/medicina62091694

**Published:** 2026-09-04

**Authors:** Derya Başak Tanburoğlu, Emrah Günay, Engin Onan, Dilek Torun, Fatih Dede, Emre Çankaya, Özkan Güngör, Feyza Nur Sarışık, İsmail Koçyiğit, Kader Kılıç, Kadir Gökhan Atılgan, Mümtaz Yılmaz, Tuncay Sahutoğlu, Mehmet Polat

**Affiliations:** 1 Department of Nephrology, Adana City Training and Research Hospital, Adana 01230, Türkiye; egnay01@gmail.com; 2 Department of Nephrology, Adana Başkent University Dr. Turgut Noyan Training and Research Hospital, Adana 01250, Türkiye; onanmd@gmail.com (E.O.); dtorun@baskent.edu.tr (D.T.); 3 Department of Nephrology, Ankara Bilkent City Hospital, Ankara 06800, Türkiye; fatihdede@sbu.edu.tr (F.D.); emrecnky@hotmail.com (E.Ç.); 4 Department of Nephrology, Kahramanmaraş Sütçü İmam University, Kahramanmaraş 46040, Türkiye; ozkangungor@yahoo.com (Ö.G.); drfeyzanur@gmail.com (F.N.S.); 5 Department of Nephrology, Erciyes University, Kayseri 38039, Türkiye; ikocyigit@erciyes.edu.tr (İ.K.); kdrklc345@gmail.com (K.K.); 6 Department of Nephrology, Ankara Etlik City Hospital, Ankara 06170, Türkiye; drgokhanatilgan@gmail.com; 7 Department of Nephrology, Ege University, İzmir 35100, Türkiye; mumtaz.yilmaz@ege.edu.tr; 8 Department of Nephrology, Mehmet Akif İnan Training and Research Hospital, Şanlıurfa 63040, Türkiye; tuncay.sahutoglu@sbu.edu.tr; 9 Department of Nephrology, Nevşehir State Hospital, Nevşehir 50100, Türkiye; mehmetpolat32@gmail.com

**Keywords:** early peritonitis, elderly patients, mortality, peritoneal dialysis, peritonitis

## Abstract

*Background and Objectives:* This study aimed to determine the incidence of peritonitis developing within the first six months of therapy in peritoneal dialysis (PD) patients aged 65 years and older, and to evaluate the risk factors associated with one-year mortality. *Materials and Methods:* A total of 227 PD patients aged ≥65 years across nine centers were evaluated in this retrospective, multicentre cohort study. Demographic characteristics, comorbidities, laboratory parameters, and clinical outcomes were analyzed using electronic health records. Early peritonitis was defined as an episode occurring within the first six months following PD initiation. Univariate and multivariable logistic regression analyses were performed to identify predictors of one-year mortality. *Results:* Early peritonitis developed in 70 patients (30.8%), and the overall one-year mortality rate was 15.9% (*n* = 36). In univariate analysis, one-year mortality was significantly associated with underlying coronary heart disease (58.3% vs. 36.1%, *p* = 0.012) and heart failure (50.0% vs. 31.9%, *p* = 0.037). The number of peritonitis episodes within the first six months was not significantly associated with 1-year mortality (*p* = 0.108). In the multivariable logistic regression model, peritonitis within the first six months was identified as an independent predictor of mortality, increasing the risk of death (OR: 11.438; 95% CI: 1.352–96.749; *p* = 0.025). Coronary heart disease, heart failure, and peak CRP during the peritonitis attack did not reach statistical significance in this model (all *p* > 0.05), though this model should be interpreted cautiously given the limited number of events (17) relative to the number of predictors (4). *Conclusions:* Peritonitis developing during the early phase of therapy is independently associated with an increased risk of one-year mortality in elderly PD patients. Mortality in this population appears to be influenced by early infectious complications alongside underlying cardiovascular comorbidity burden. These findings highlight the clinical importance of close surveillance, early infection prophylaxis, and vigilant therapeutic strategies during the initial months of peritoneal dialysis.

## 1. Introduction

With the global aging of the population, the proportion of elderly patients diagnosed with end-stage renal failure (ESRF) has been steadily increasing [1]. This demographic shift means that the effectiveness and safety of renal replacement therapy options are becoming increasingly important for elderly patients. PD is considered a preferred treatment option for patients aged 65 years and older due to its better hemodynamic stability compared with hemodialysis (HD), preservation of residual renal function, and improvement in patients’ quality of life [2,3]. However, despite advances in connection technologies, peritonitis remains the most serious complication of peritoneal dialysis (PD) and represents an independent risk factor for hospitalization, technique failure, and patient mortality [4,5]. Current literature suggests that the timing and type of peritonitis episodes are key determinants of clinical outcomes in patients undergoing peritoneal dialysis. In particular, infections occurring within the first 6 months of therapy, defined as early-onset peritonitis (EOP), are commonly associated with inadequate patient technique (unskilled manipulation) or insufficient training [4]. As emphasised in the 2022 updated guidelines of the International Society for Peritoneal Dialysis (ISPD), the standardisation of definitions and outcomes relating to peritonitis is of critical importance for improving clinical practice and predicting prognosis [5]. The management of peritonitis in elderly patients is more complex due to the specific physiological and clinical characteristics of this group. The incidence of peritonitis has been reported to be higher in individuals aged 65 years and older compared with younger patients [6,7]. In this population, factors such as diminished visual acuity can hinder the detection of cloudy dialysate—the earliest sign of peritonitis—thereby leading to diagnostic delays. Additionally, hypoalbuminemia, hypokalemia, and general frailty predispose elderly patients to infections, thereby elevating mortality risks [1,5]. Studies indicate that the risk of peritonitis-related mortality in elderly patients is approximately 2.3 times higher than in the younger population [8,9,10]. Investigations into the long-term effects of EOP show that it independently predicts patient mortality, in addition to compromising technique survival [11]. The aim of this study was to evaluate how peritonitis attacks developing during the initial 6 months of treatment affect the 1-year mortality rates of elderly peritoneal dialysis patients aged ≥65 years.

## 2. Methods

### 2.1. Study Design and Patients

**Figure 1:**
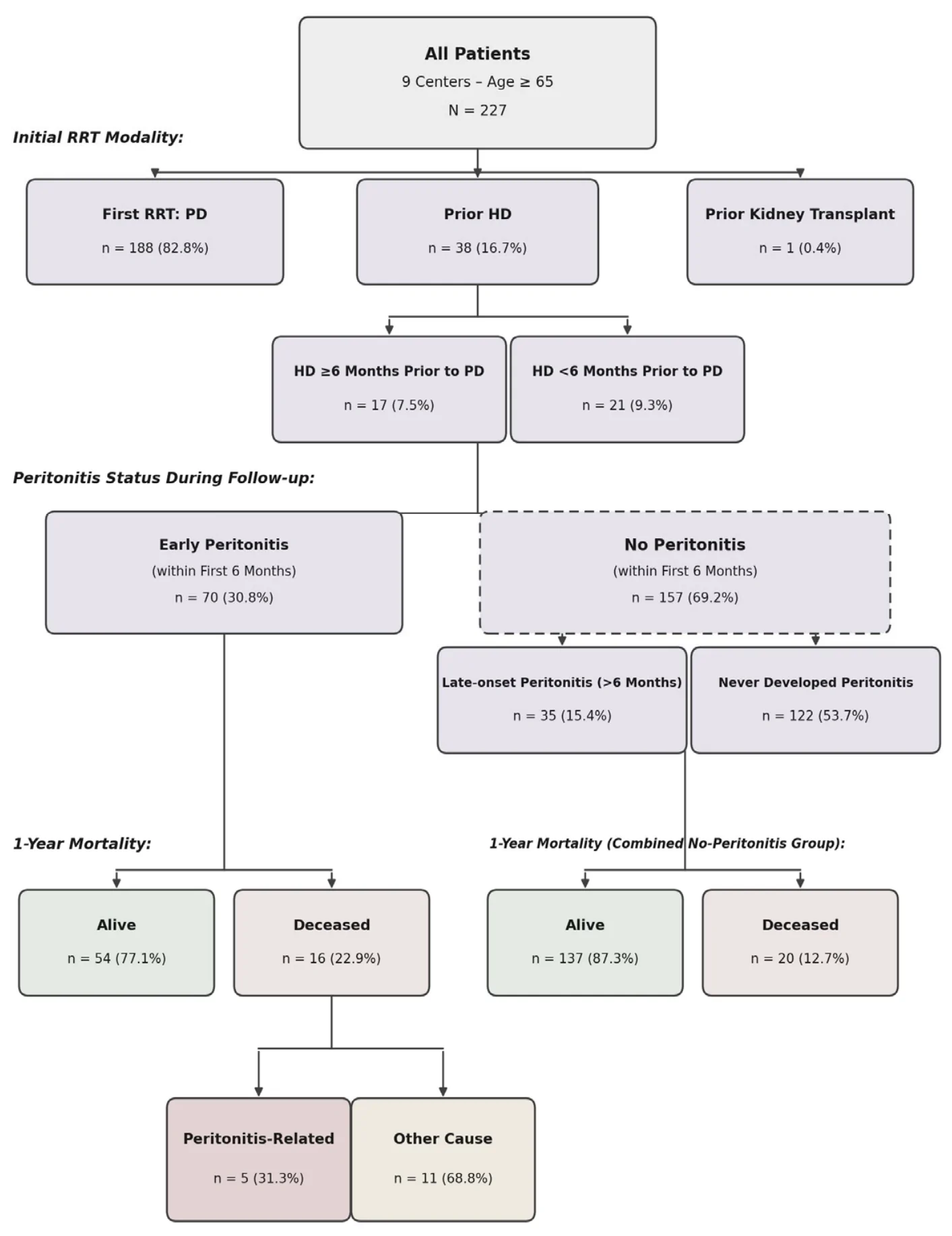
Flowchart of study protocol. Green boxes indicate patients who were alive at 1 year, and pink boxes indicate patients who died within 1 year. The dashed-border box denotes the combined “No Peritonitis” group, encompassing both patients with late-onset peritonitis and those who never developed peritonitis during follow-up.

This retrospective, multicenter cohort study included peritoneal dialysis patients aged ≥65 years who were monitored at nine distinct centers in Türkiye between 2015 and 2025. From the onset of PD, all patients were monitored until the occurrence of death, switching to HD, renal transplantation, or study termination. Data on demographics, age, sex, ESRD etiology, prior renal replacement therapy, comorbidities, medications, and laboratory profiles were extracted from patient files and electronic health records. Patients were excluded if they were younger than 65 years of age, had insufficient clinical or follow-up data, or underwent PD catheter placement but never initiated peritoneal dialysis (Figure 1).

The study was conducted in accordance with the Declaration of Helsinki and approved by the Scientific Research Ethics Committee of Adana City Training and Research Hospital (Meeting No. 21, Date: 15 January 2026, Decision No. 990).

### 2.2. Definitions

#### 2.2.1. Diagnosis of Peritonitis

Diagnosis of peritonitis was based on the International Society for Peritoneal Dialysis (ISPD) guidelines if two or more of the following criteria were present: (1) clinical features consistent with peritonitis (e.g., abdominal pain and/or cloudy dialysate effluent); (2) dialysate white blood cell (WBC) count > 100 cells/µL (after a dwell time of ≥2 h) with >50% polymorphonuclear leukocytes; and (3) positive dialysate culture.

#### 2.2.2. Early-Onset Peritonitis and Mortality Definitions

Early-onset peritonitis was defined as the first peritonitis attack within 6 months post-PD initiation. One-year mortality was defined as death from any cause within 12 months after the onset of PD treatment. Patients were followed for a minimum of 12 months from PD initiation to ascertain one-year mortality status. Patients who died within this period—including those who developed early peritonitis—were followed until the date of death, whereas surviving patients, regardless of peritonitis status, were required to have completed at least 12 months of follow-up to be included in the mortality analysis. In accordance with International Society for Peritoneal Dialysis (ISPD) guidelines, peritonitis-related mortality was defined as death occurring during an active peritonitis episode or within 30 days of peritonitis onset due to direct peritonitis-related complications.

#### 2.2.3. Polypharmacy

Polypharmacy was defined as the concomitant use of five or more medications by a patient.

#### 2.2.4. Treatment Outcome Definitions

The definition of treatment success was according to the 2022 guidelines of the International Society for Peritoneal Dialysis (ISPD). Cure was defined as clinical resolution of peritonitis without removal of catheter or death. Refractory peritonitis was defined as persistence of cloudy dialysate effluent after 5 days of appropriate antibiotics and was an indication for catheter removal. Relapsing peritonitis referred to an episode occurring within 4 weeks of completing treatment for a prior episode, caused by the same organism or resulting in a sterile culture. Recurrent peritonitis was defined as an episode occurring within 4 weeks of completing therapy but caused by a different organism, whereas repeat peritonitis was defined as an episode occurring more than 4 weeks after completing therapy, caused by the same organism. Permanent transfer to hemodialysis (technique failure) was defined as catheter removal due to peritonitis without subsequent re-implantation, resulting in long-term hemodialysis.

### 2.3. Treatment, Clinical Management and Laboratory Measurements

#### 2.3.1. Initial Patient Presentation and Antibiotic Treatment Location

All patients presented directly to their primary outpatient peritoneal dialysis units upon symptom onset. Empirical antibiotic therapy was initiated exclusively at these centers following initial clinical evaluation and dialysate sampling. No patients had been managed by private practices or received empirical antibiotic treatment at outside healthcare facilities prior to presentation.

#### 2.3.2. Peritonitis Management Protocol

All patients were monitored in accordance with standard PD protocols. Patients who developed peritonitis were evaluated immediately, and empirical intraperitoneal antibiotic therapy was initiated; treatment was subsequently adjusted based on culture results and antimicrobial susceptibility testing. Hospitalization decisions, the duration of antibiotic therapy, catheter replacement, and the need for transition to hemodialysis were determined based on the patient’s clinical status and at the discretion of the attending physician. Peritonitis management was carried out in accordance with the ISPD 2022 Peritonitis Guidelines, specifically following their recommendations regarding empirical antibiotic selection to cover both Gram-positive and Gram-negative organisms pending culture results, the preferential use of the intraperitoneal route for antibiotic administration, reassessment of clinical response within the first 2–3 days of therapy, and de-escalation or modification of therapy once culture and susceptibility results became available; in addition, per ISPD guidance, patients who were hemodynamically stable and had no evidence of systemic infection were treated as outpatients. Dialysate effluent samples were obtained for leukocyte cell count, differential and microbiologic cultures prior to initiation of empiric therapy. Empiric intraperitoneal (IP) antibiotics were started immediately, with cefazolin or vancomycin for Gram-positive coverage and ceftazidime for Gram-negative coverage, according to the local microbiologic flora and resistance patterns of each center. Patients were reassessed within 72 h of starting treatment, and antibiotic therapy was adjusted according to definitive culture and susceptibility results. Perioperative intravenous antibiotic prophylaxis was routinely given before PD catheter placement in all centers.

#### 2.3.3. Laboratory Measurements

Baseline laboratory parameters, including serum albumin, hemoglobin, and estimated glomerular filtration rate (eGFR), were obtained at the time of PD initiation. eGFR was calculated using a serum creatinine-based formula. In patients who developed peritonitis, C-reactive protein (CRP) was recorded as the highest value measured at any point during the peritonitis attack; this attack-time CRP value is therefore only available for patients who experienced a peritonitis episode and reflects a different timepoint than the PD-initiation baseline parameters.

#### 2.3.4. Catheter Replacement and Technique Failure

Catheter replacement was defined as a two-stage procedure involving the removal of the existing peritoneal dialysis catheter due to peritonitis, followed by temporary hemodialysis (HD) until complete clinical resolution of the infection, after which a new catheter was implanted in a subsequent surgical session. Technique failure was defined as permanent catheter removal with transition to long-term HD without return to peritoneal dialysis.

### 2.4. Statistical Analysis

Statistical analyses were performed using the IBM SPSS 20 software package (IBM Corp., Armonk, NY, USA). The normality of continuous variables was assessed using the Kolmogorov–Smirnov test; data following a normal distribution are expressed as mean ± standard deviation, while nonnormally distributed data are expressed as median (Q1–Q3). For comparisons between two independent groups, Student’s *t*-test was used for normally distributed variables, and the Mann–Whitney U test was used for non-normally distributed variables. Categorical variables are presented as counts and percentages; the chi-square test was used for intergroup comparisons, and the Fisher exact test was used when the expected cell count was insufficient. In univariate analyses, *p* < 0.05 was considered statistically significant; *p* < 0.25 was used as the cutoff value and clinical significance criterion for determining candidate variables to be included in the multivariate model. Multivariate logistic regression analysis was performed to identify independent predictors of one-year mortality, and the model was constructed using the Forward Likelihood Ratio method. Multicollinearity among variables was assessed using the Variance Inflation Factor (VIF). Results are reported as odds ratios and 95% confidence intervals, and a two-tailed *p* < 0.05 value was considered significant in all analyses.

## 3. Results

A total of 227 patients from nine participating centers were included. Overall, 70 patients (30.8%) reported peritonitis in the first six months. The largest cohort was found in Adana Başkent University Dr. Turgut Noyan Training and Research Hospital (*n* = 60, 26.4%), followed by Erciyes University (*n* = 59, 26.0%), Adana City Training and Research Hospital (*n* = 45, 19.8%), Ankara Bilkent City Hospital (*n* = 21, 9.3%), Ankara Etlik City Hospital (*n* = 10, 4.4%), Nevşehir State Hospital (*n* = 10, 4.4%), Mehmet Akif İnan Training and Research Hospital (*n* = 10, 4.4%), Kahramanmaraş Sütçü İmam University (*n* = 6, 2.6%), and Ege University (*n* = 6, 2.6%). The incidence of peritonitis in the first 6 months ranged from 10.0% (*n* = 1/10) at Mehmet Akif İnan Training and Research Hospital to 60.0% (*n* = 6/10) at Nevşehir State Hospital. Erciyes University had the highest absolute number of peritonitis cases (*n* = 29/59, 49.2%).

Peritoneal dialysis served as the initial renal replacement therapy (RRT) modality in 188 patients (82.8%). A history of maintenance hemodialysis (HD) for more than six months prior to PD initiation was reported in 17 patients (7.5%), a further 21 patients (9.3%) had received HD for less than six months prior to PD, and 1 patient (0.4%) had a history of kidney transplantation prior to PD (17 + 21 + 1 = 39 patients who did not start on PD as their first RRT modality, together with the 188 PD-first patients accounting for the full cohort of 227) (Table 1). Among the 197 patients with available data on this variable (30 of the 227 patients had missing data), 22 (11.2%) transitioned from PD to hemodialysis; among the 105 patients who developed peritonitis at some point during follow-up (70 early-onset and 35 late-onset), this transition occurred in 9 of the 70 patients (12.9%) with early-onset peritonitis and in 7 of the 35 patients (20.0%) with late-onset peritonitis. Catheter replacement (as defined in Section 2.3.4) was performed in 10 patients overall among the 105 patients who developed peritonitis (complete data available for all 105 patients); when stratified by timing of onset, catheter replacement occurred in 3 of the 70 patients (4.3%) with early-onset peritonitis and in 7 of the 35 patients (20.0%) with late-onset peritonitis. Evaluation of baseline comorbidities revealed that diabetes mellitus was present in 127 patients (55.9%), hypertension in 194 patients (85.5%), heart failure in 79 patients (34.8%), coronary heart disease in 90 patients (39.6%), and cancer in 13 patients (7.1%). Polypharmacy, defined as the concurrent use of five or more medications, was documented in 184 patients (81.1%). Within the 12-month follow-up period, all-cause mortality occurred in 36 patients (15.9%), while 191 patients (84.1%) survived.

**Immunosuppression status:** Regarding baseline immunosuppressive conditions, 13 patients had a history of malignancy, 6 of whom had received chemotherapy within the last year. Additionally, 1 patient had a history of renal transplantation and was receiving methylprednisolone therapy; this was the same patient noted above with a prior kidney transplant as the initial RRT modality. Neither malignancy history nor immunosuppressive therapy was significantly associated with peritonitis development or 1-year mortality in univariate analysis (Table 2 and Table 3).

When peritoneal dialysis modalities were examined, it was found that the vast majority of the study population was receiving continuous ambulatory peritoneal dialysis (CAPD; *n* = 218), while 9 patients were on automated peritoneal dialysis (APD). Among the CAPD group, 127 patients (58.3%) were male and 91 (41.7%) were female; among the APD group, 4 patients (44.4%) were male and 5 (55.6%) were female. Of the 22 patients who transitioned from PD to hemodialysis, 2 underwent permanent transition (technique failure) without subsequent return to peritoneal dialysis. Laboratory investigations demonstrated a median serum albumin level of 3.40 g/dL (interquartile range [IQR]: 3.20–3.80 g/dL), a mean hemoglobin level of 10.98 ± 1.61 g/dL, and a median estimated glomerular filtration rate (eGFR) of 10.50 mL/min (IQR: 8.00–14.50 mL/min; valid data available for all 227 patients) (Table 1). Peritonitis-related mortality according to ISPD criteria occurred in 5 patients, all of whom had early-onset peritonitis. Additionally, 31 patients died from non-peritonitis causes within 12 months following PD initiation. Microbiological culture results were analyzed to characterize the pathogen spectrum across centers. Because a dialysate culture can only be obtained during an active peritonitis episode, these results were analyzed exclusively among the 105 patients who developed peritonitis at some point during follow-up, rather than the full cohort of 227; valid culture data were available for 96 of these 105 patients (9 missing). Among these, the majority of cases were culture-negative (*n* = 61, 63.5%). Among the culture-positive cases, Gram-positive microorganisms were the predominant isolated pathogens (*n* = 27, 28.1%), followed by Gram-negative organisms (*n* = 5, 5.2%). Polymicrobial growth was observed in 3 patients (3.1%), whereas no cases of fungal peritonitis were identified (*n* = 0, 0.0%). When stratified by timing of onset, the pathogen distribution differed between early- and late-onset peritonitis episodes: among the 70 early-onset episodes (within the first 6 months), valid culture data were available for 67 (3 missing); culture-negative results predominated (*n* = 49, 73.1%), followed by Gram-positive (*n* = 13, 19.4%), polymicrobial (*n* = 3, 4.5%), and Gram-negative (*n* = 2, 3.0%) organisms. Among the 35 late-onset episodes (after 6 months), valid culture data were available for 29 (6 missing); Gram-positive organisms were more frequent (*n* = 14, 48.3%), followed by culture-negative (*n* = 12, 41.4%) and Gram-negative (*n* = 3, 10.3%) results. Among the 17 patients who died and had experienced peritonitis at some point during follow-up, valid culture data were available for all 17 (no missing data); culture-negative peritonitis was most common (*n* = 10, 58.8%), followed by Gram-positive (*n* = 4, 23.5%), Gram-negative (*n* = 2, 11.8%), and polymicrobial (*n* = 1, 5.9%) organisms. Treatment outcomes were categorized according to the ISPD definitions outlined in Section 2.2.4. Among the 105 peritonitis episodes, outcome data were available for 101 (4 missing): cure was achieved in 77 episodes (76.2%), while 11 (10.9%) were classified as refractory, 6 (5.9%) as relapsing, 6 (5.9%) as repeat, and 1 (1.0%) as recurrent peritonitis. Of the 105 patients who developed peritonitis, 71 (67.6%) were managed with inpatient treatment, while the remaining 34 (32.4%) were managed as outpatients, consistent with the criteria described in Section 2.3.2 (hemodynamically stable patients with no evidence of systemic infection were treated as outpatients).

**Table 1:** Clinical characteristics of the patients.

		All Patients (*n* = 227)
**Gender**	Male	131 (57.7)
	Female	96 (42.3)
**PD Modality**	CAPD	218 (96.0)
	APD	9 (4.0)
**First RRT peritoneal dialysis**	Yes	188 (82.8)
	No	39 (17.2)
**HD for more than 6 months prior to PD**	Yes	17 (7.5)
	No	210 (92.5)
**HD for less than 6 months prior to PD**	Yes	21 (9.3)
	No	206 (90.7)
**Kidney transplant prior to PD**	Yes	1 (0.4)
	No	226 (99.6)
**Peritonitis within the first 6 months**	Yes	70 (30.8)
	No	157 (69.2)
Catheter replacement *	Yes	10 (9.5)
	No	95 (90.5)
Switch from PD to HD *	Yes	22 (11.2)
	No	175 (88.8)
**Diabetes Mellitus**	Yes	127 (55.9)
	No	100 (44.1)
**Hypertension**	Yes	194 (85.5)
	No	33 (14.5)
**Heart failure**	Yes	79 (34.8)
	No	148 (65.2)
**Coronary heart disease**	Yes	90 (39.6)
	No	137 (60.4)
Cancer *	Yes	13 (7.1)
	No	170 (92.9)
**Polypharmacy (use of 5 or more medications)**	Yes	184 (81.1)
	No	43 (18.9)
**Mortality within 12 months**	Alive	191 (84.1)
	Ex	36 (15.9)
**Albumin (g/dL)**		3.40 (3.20–3.80)
**Hemoglobin (g/dL)**		10.98 ± 1.61
**e-GFR (mL/min)**		10.50 (8.00–14.50)

Categorical variables are presented as *n* (%), while numerical variables are presented as mean ± SD or median (Q1–Q3) according to the distribution type. * Percentages for catheter replacement are calculated among the 105 patients who developed peritonitis (complete data available for all); percentages for switch from PD to HD and cancer are calculated among patients with available data for each variable (*n* = 197 and *n* = 183, respectively); data were missing or not recorded for the remaining patients. PD: peritoneal dialysis; RRT: renal replacement therapy; HD: hemodialysis; e-GFR: estimated glomerular filtration rate.

When demographic, clinical, and laboratory variables were evaluated according to the development of peritonitis within the first six months, no statistically significant differences were observed between the groups regarding age, peritoneal dialysis as the initial renal replacement therapy, history of prior hemodialysis, renal transplantation history, diabetes mellitus, hypertension, heart failure, coronary artery disease, history of malignancy, polypharmacy status, serum albumin, or hemoglobin levels (all *p* > 0.05) (Table 2).

**Table 2:** Demographic and clinical variables affecting the development of peritonitis in the first six months.

		Peritonitis in the First 6 Months (*n* = 70)	No Peritonitis in the First 6 Months (*n* = 157)	*p*
**Age (years), mean ± SD**		72.8 ± 5.6	73.4 ± 5.6	0.416
**First RRT peritoneal dialysis**	Yes	59 (84.3%)	129 (82.2%)	0.696
	No	11 (15.7%)	28 (17.8%)	
**HD for >6 months prior to PD**	Yes	5 (7.1%)	12 (7.6%)	0.896
	No	65 (92.9%)	145 (92.4%)	
**Kidney transplant prior to PD**	Yes	0 (0.0%)	1 (0.6%)	0.503
	No	70 (100.0%)	156 (99.4%)	
**Diabetes Mellitus**	Yes	39 (55.7%)	88 (56.1%)	0.962
	No	31 (44.3%)	69 (43.9%)	
**Hypertension**	Yes	62 (88.6%)	132 (84.1%)	0.375
	No	8 (11.4%)	25 (15.9%)	
**Heart failure**	Yes	22 (31.4%)	57 (36.3%)	0.476
	No	48 (68.6%)	100 (63.7%)	
**Coronary heart disease**	Yes	29 (41.4%)	61 (38.9%)	0.714
	No	41 (58.6%)	96 (61.1%)	
Cancer *	Yes	2 (3.4%)	11 (8.9%)	0.229
	No	57 (96.6%)	113 (91.1%)	
**Polypharmacy (≥5 medications)**	Yes	58 (82.9%)	126 (80.3%)	0.644
	No	12 (17.1%)	31 (19.7%)	
**Albumin (g/dL)**		3.40 (3.19–3.90)	3.40 (3.20–3.70)	0.453
**Hemoglobin (g/dL)**		11.09 ± 1.55	10.93 ± 1.64	0.483
**e-GFR (mL/min)**		11.00 (8.85–21.00)	10.00 (8.00–13.00)	0.064

* Analysis for cancer was performed among patients with available data (*n* = 183).

In the 1-year mortality assessment, no statistically significant differences were observed between survivors and non-survivors regarding age, gender, initial renal replacement therapy modality, prior hemodialysis history, renal transplantation status, diabetes mellitus, hypertension, history of malignancy, polypharmacy status, or laboratory parameters (serum albumin, hemoglobin, eGFR) (all *p* > 0.05). Furthermore, peritonitis management outcomes, including inpatient hospitalization, catheter replacement, and permanent transfer to hemodialysis, were not significantly associated with mortality (all *p* > 0.05). In contrast, underlying cardiovascular comorbidities were significantly associated with mortality. Non-survivors exhibited a significantly higher prevalence of coronary heart disease (58.3% vs. 36.1%, *p* = 0.012) and heart failure (50.0% vs. 31.9%, *p* = 0.037) compared to survivors. However, the number of peritonitis episodes within the first six months was not significantly associated with 1-year mortality (*p* = 0.108) (Table 3).

**Table 3:** Risk Factors for 1-Year Mortality in Peritoneal Dialysis Patients.

		1-Year Mortality Alive (*n* = 191)	1-Year Mortality Exitus (*n* = 36)	*p*
**Age (years), mean ± SD**		73.2 ± 5.6	73.4 ± 6.0	0.865
**Gender**	Male	112 (58.6%)	19 (52.8%)	0.514
	Female	79 (41.4%)	17 (47.2%)	
**First RRT peritoneal dialysis**	Yes	159 (83.2%)	29 (80.6%)	0.638
	No	32 (16.8%)	7 (19.4%)	
**HD for >6 months prior to PD**	Yes	13 (6.8%)	4 (11.1%)	0.321
	No	178 (93.2%)	32 (88.9%)	
**Kidney transplant prior to PD**	Yes	1 (0.5%)	0 (0.0%)	0.663
	No	190 (99.5%)	36 (100.0%)	
**Peritonitis within the first 6 months**	Yes	54 (28.3%)	16 (44.4%)	0.054
	No	137 (71.7%)	20 (55.6%)	
**Number of peritonitis in 6 months**	None (0)	137 (60.4%)	20 (8.8%)	0.108
	Single (1)	41 (18.1%)	13 (5.7%)	
	Two or more (≥2)	13 (5.7%)	3 (1.3%)	
Catheter replacement *	Yes	8 (9.1%)	2 (11.8%)	0.663
	No	80 (90.9%)	15 (88.2%)	
**Switch from PD to HD**	Yes	17 (10.2%)	5 (16.1%)	0.339
	No	149 (89.8%)	26 (83.9%)	
**Diabetes Mellitus**	Yes	105 (55.0%)	22 (61.1%)	0.496
	No	86 (45.0%)	14 (38.9%)	
**Hypertension**	Yes	166 (86.9%)	28 (77.8%)	0.154
	No	25 (13.1%)	8 (22.2%)	
**Heart failure**	Yes	61 (31.9%)	18 (50.0%)	0.037
	No	130 (68.1%)	18 (50.0%)	
**Coronary heart disease**	Yes	69 (36.1%)	21 (58.3%)	0.012
	No	122 (63.9%)	15 (41.7%)	
Cancer *	Yes	10 (6.3%)	3 (12.5%)	0.384
	No	149 (93.7%)	21 (87.5%)	
**Polypharmacy (≥5 medications)**	Yes	151 (79.1%)	33 (91.7%)	0.077
	No	40 (20.9%)	3 (8.3%)	
**Albumin (g/dL)**		3.40 (3.20–3.80)	3.32 (3.18–3.85)	0.335
**Hemoglobin (g/dL)**		10.94 ± 1.62	11.21 ± 1.59	0.353
**e-GFR (mL/min)**		10.50 (8.00–14.50)	10.50 (7.25–15.75)	0.852

* Analyses for cancer (*n* = 183) and catheter replacement (*n* = 105) were performed among patients with available data. Cancer status was known for 183 patients (13 with and 170 without a history of malignancy); cancer screening had not been performed on the remaining 44 patients, for whom no information was available.

Given that Table 2 compared early-onset peritonitis against a heterogeneous reference group combining late-onset and no-peritonitis patients, we additionally performed a direct comparison between early-onset (≤6 months) and late-onset (>6 months) peritonitis (Table 4).

Variables that were found to be significant in univariate analysis or considered clinically relevant—including peritonitis within the first six months, hospitalization status, catheter replacement, transition from PD to hemodialysis, coronary heart disease, baseline hemoglobin level, age, and gender—were evaluated for inclusion in the multivariable logistic regression model. Multicollinearity among variables was assessed, and no collinearity issues were detected (all VIFs < 2). In the final multivariable regression model, which included peritonitis within the first six months, coronary heart disease, heart failure, and peak CRP during the peritonitis attack (*n* = 105 patients with complete data on all four variables; 17 mortality events), the development of peritonitis within the first six months remained the only independent predictor of mortality, with a markedly increased risk of death (OR: 11.438; 95% CI: 1.352–96.749; *p* = 0.025). Coronary heart disease (OR: 1.273; 95% CI: 0.366–4.429; *p* = 0.704), heart failure (OR: 1.240; 95% CI: 0.336–4.578; *p* = 0.747), and peak CRP (OR: 1.006; 95% CI: 0.999–1.012; *p* = 0.072) did not reach statistical significance in this model. Given the limited number of events relative to the number of predictors (17 events for 4 variables, below the conventional rule-of-thumb of ≥10 events per variable), these multivariable estimates—particularly the wide confidence interval around the peritonitis odds ratio—should be interpreted with caution and considered exploratory rather than definitive (Table 5).

## 4. Discussion

This study demonstrates the critical impact of peritonitis episodes occurring within the first 6 months of therapy on 1-year mortality rates and technique survival among peritoneal dialysis patients aged 65 years and older. Our results reveal that early peritonitis is highly prevalent in this population (30.8%) and is strongly associated with an increased risk of mortality. Particularly during the initial 6 months, the substantial elevation of mortality risk associated with peritonitis suggests that this timeframe constitutes a critical prognostic window for older peritoneal dialysis patients. Underlying cardiovascular comorbidities—specifically coronary heart disease and heart failure—were also significantly associated with mortality in univariate analysis, though neither remained independently significant after adjustment, likely reflecting the substantial overlap between these two cardiovascular conditions rather than a true absence of effect. Consequently, these data suggest that early peritonitis should not be viewed simply as an isolated complication, but as a major clinical indicator forecasting mortality in older patients.

Although the literature exhibits a wide divergence regarding the definition of EOP, ranging from 3 to 24 months, numerous contemporary studies along with our study data support the first 6 months as the critical threshold [12,13,14]. In an analysis conducted by Feng et al. (2016), patients experiencing EOP were found to have a significantly higher risk of mortality (HR: 2.03) and rate of technique failure (HR: 1.69) [11]. Similarly, studies across diverse cohorts demonstrate that peritonitis occurs more frequently during the early phase of treatment, with the initial peritonitis episode commonly manifesting within the first 6 months [15,16]. Particularly in elderly patients, an infection occurring during the initial months following dialysis initiation is hypothesized to cause irreversible damage to peritoneal membrane functions and compromise the patient’s overall health status, thereby paving the way for fatal outcomes within a 1-year period [11]. Long-term analyses conducted by Nardelli et al. (2024) also confirm that peritonitis is the most common cause of permanent transition to hemodialysis and is directly associated with mortality [4]. A study targeting the elderly population by Wang et al. (2015) supports our core hypothesis, confirming that peritonitis episodes occurring within the first 6 months are associated with significantly higher mortality rates compared with late-onset episodes [17]. While the findings obtained in our study are consistent with the existing literature, they reveal noteworthy aspects specific to our elderly population. Specifically, multivariable logistic regression, adjusting for coronary heart disease, heart failure, and peak CRP, demonstrated a markedly increased mortality risk associated with early peritonitis (OR: 11.438; 95% CI: 1.352–96.749; *p* = 0.025), although this estimate should be interpreted cautiously given the limited number of events relative to the number of predictors in this model (Table 5). This phenomenon can be attributed to the heightened vulnerability to infections, immunosenescence, and a greater burden of comorbidities in elderly patients. Furthermore, as documented in the literature, the association of early-onset peritonitis episodes with higher subsequent peritonitis rates [12] and their capacity to induce irreversible alterations in peritoneal membrane functions [18,19] can adversely impact long-term clinical outcomes.

Catheter-related infections play a pivotal role in the development of peritonitis, and the appropriate prevention and management of exit-site infections, in particular, is recognized as a critical step in reducing the overall infectious burden [20]. Current clinical practice guidelines aim to minimize the incidence of catheter exit-site infections and provide various recommendations regarding prophylactic strategies [21]. Studies evaluating catheter outcomes have demonstrated that infectious complications are among the leading causes of catheter loss and significantly impact technique survival. Indeed, a cohort study involving 340 patients undergoing PD catheter implantation demonstrated that infections and mechanical dysfunctions were among the most common reasons for catheter removal; furthermore, postoperative revision was identified as an independent risk factor adversely affecting technique survival [22]. However, an evaluation of data regarding catheter reinsertion suggests that the specific reason for initial catheter removal may influence subsequent clinical outcomes. In a single-center study, it was reported that technique survival following a subsequent trial of peritoneal dialysis was superior in patients whose catheters were removed due to infection compared with those removed for mechanical reasons. Furthermore, no statistically significant difference in survival was detected between patients who attempted PD resumption and those who transitioned to hemodialysis [23]. In alignment with these data, although catheter replacement was required in 9.5% of patients in our study due to refractory peritonitis, catheter replacement itself was not significantly associated with 1-year mortality (*p* = 0.663). This suggests that timely catheter removal and appropriate clinical intervention can effectively mitigate the fatal consequences of severe infectious episodes.

Our microbiological findings also carry clinical relevance. Culture-negative results predominated across the peritonitis episodes in our cohort and remained common even among patients who ultimately died (58.8%), although somewhat less frequent than in the overall peritonitis cohort (63.5%) or among early-onset episodes specifically (73.1%). While culture-negative peritonitis is generally associated with a more favorable prognosis in the broader PD literature, its high frequency in our elderly population may instead reflect prior antibiotic exposure, fastidious or slow-growing organisms, or suboptimal culture technique rather than a truly less virulent process; the elevated mortality we observed despite this culture-negative predominance suggests that clinicians should not interpret a negative culture as reassurance against a severe clinical course in older PD patients, and that empirical antibiotic coverage should continue to be guided by clinical severity rather than culture positivity alone. Similarly, the higher prevalence of Gram-positive organisms among late-onset episodes (48.3%) compared with early-onset episodes (19.4%) may reflect a shift in exposure route over time on PD (e.g., increasing relative contribution of touch-contamination and exit-site-related infections as opposed to perioperative or early post-implantation events), a hypothesis that merits prospective investigation.

This study has several limitations that warrant consideration. First, its retrospective, multicentre design may introduce record-based measurement variation across participating centers, and despite statistical adjustment, residual confounding from unmeasured variables cannot be entirely excluded. Second, our multivariable model was constrained by a limited number of mortality events relative to the number of predictors (17 events for 4 variables, below the conventional guideline of ≥10 events per variable); the correspondingly wide confidence interval around the peritonitis odds ratio indicates that this estimate should be considered exploratory rather than definitive, and warrants confirmation in larger cohorts. Third, because peak CRP during the peritonitis attack is, by definition, only measurable in patients who experienced an episode, its inclusion as a covariate structurally restricted the multivariable analysis to the subset of patients with peritonitis, limiting our ability to compare peritonitis against a fully independent, non-peritonitis reference group within the same model. Fourth, serial inflammatory markers (e.g., white blood cell counts before and after treatment) were not uniformly available across centers, precluding assessment of early treatment response as a prognostic factor. Fifth, patient-level ISPD outcome categorization (cure, relapse, recurrence, refractory disease) relied on retrospective chart review rather than prospective, standardized adjudication, which may be subject to documentation variability across centers. Finally, our cohort was drawn exclusively from Turkish centers, which may limit generalizability to populations with different healthcare infrastructure, antibiotic resistance patterns, or patient demographics. Nonetheless, a strength of this investigation is its multicentre design, utilizing data from nine centers to evaluate an elderly cohort, providing multicentre evidence on early clinical trajectories and risk factors in this population.

## 5. Conclusions

In conclusion, this multicentre study suggests that early-onset peritonitis occurring within the first six months of peritoneal dialysis is associated with an increased risk of 1-year mortality in patients aged 65 years and older. Patient outcomes in this population appear to be influenced by both early infectious complications and underlying cardiovascular comorbidity burden. These findings highlight the potential importance of enhanced clinical surveillance, early infection prophylaxis, and careful monitoring during the initial months of therapy. Further prospective studies are needed to clarify management strategies and optimize outcomes in older adults initiating peritoneal dialysis.

## Figures and Tables

**Table 4:** Comparison between early-onset (≤6 months) and late-onset (>6 months) peritonitis.

Variable	Early Peritonitis (≤6 Months) (*n* = 70)	Late Peritonitis (>6 Months) (*n* = 35)	*p*
**Age (years), mean ± SD**	72.8 ± 5.6	73.4 ± 4.9	0.567
**Gender, Male**	41 (58.6%)	20 (57.1%)	0.889
**Diabetes Mellitus**	39 (55.7%)	20 (57.1%)	0.889
**Hypertension**	62 (88.6%)	32 (91.4%)	0.748
**Heart failure**	22 (31.4%)	8 (22.9%)	0.359
**Coronary heart disease**	29 (41.4%)	7 (20.0%)	**0.029**
**Polypharmacy (≥5 medications)**	58 (82.9%)	20 (57.1%)	**0.004**
**e-GFR (mL/min), median (IQR)**	11.00 (8.85–21.00)	10.00 (8.25–12.00)	0.078

Categorical variables are presented as *n* (%); numerical variables as mean ± SD or median (IQR). Fisher’s exact test was used instead of the chi-square test for the Hypertension row (expected cell count < 5). Bold values indicate statistical significance (*p* < 0.05).

**Table 5:** Logistic regression analysis results for determining 1-year mortality.

	OR	95% CI for OR	*p*
Peritonitis within the first 6 months	11.438	1.352–96.749	**0.025**
Coronary heart disease	1.273	0.366–4.429	0.704
Heart failure	1.240	0.336–4.578	0.747
Highest CRP value during attack	1.006	0.999–1.012	0.072

OR: odds ratio, CI: confidence interval. Model included *n* = 105 patients with complete data on all four variables (17 mortality events). Given the limited number of events relative to the number of predictors (17 events for 4 variables), these estimates—particularly the wide confidence interval around the peritonitis odds ratio—should be interpreted with caution and considered exploratory rather than definitive. Bold values indicate statistical significance (*p* < 0.05).

## Data Availability

The data presented in this study are available on request from the corresponding author due to privacy and ethical restrictions. The data are not publicly available to protect patient confidentiality.

## References

[1] Sakurada T., Miyazaki M., Nakayama M., Ito Y. (2024). Peritoneal dialysis-related infections in elderly patients. Clin. Exp. Nephrol..

[2] Brown E.A., Johansson L., Farrington K., Gallagher H., Sensky T., Gordon F., Da Silva-Gane M., Beckett N., Hickson M. (2010). Broadening options for long-term dialysis in the elderly (BOLDE): Differences in quality of life on peritoneal dialysis compared to haemodialysis for older patients. Nephrol. Dial. Transplant..

[3] Brown E.A., Blake P.G., Boudville N., Davies S., de Arteaga J., Dong J., Finkelstein F., Foo M., Hurst H., Johnson D.W. (2020). International Society for Peritoneal Dialysis practice recommendations: Prescribing high-quality goal-directed peritoneal dialysis. Perit. Dial. Int..

[4] Nardelli L., Scalamogna A., Ponzano F., Sikharulidze A., Tripodi F., Vettoretti S., Alfieri C., Castellano G. (2024). Peritoneal dialysis related peritonitis: Insights from a long-term analysis of an Italian center. BMC Nephrol..

[5] Li P.K., Chow K.M., Cho Y., Fan S., Figueiredo A.E., Harris T., Kanjanabuch T., Kim Y.-L., Madero M., Malyszko J. (2022). ISPD peritonitis guideline recommendations: 2022 update on prevention and treatment. Perit. Dial. Int..

[6] Masakane I., Taniguchi M., Nakai S., Tsuchida K., Wada A., Ogata S., Hasegawa T., Hamano T., Hanafusa N. (2018). Annual dialysis data report 2016, JSDT Renal Data Registry. Ren. Replace Ther..

[7] Jiang C., Zheng Q. (2022). Outcomes of peritoneal dialysis in elderly vs non-elderly patients: A systemic review and meta-analysis. PLoS ONE.

[8] Oliver M.J., Abra G., Béchade C., Brown E., Sanchez-Escuredo A., Johnson D.W., Guedes A.M., Graham J., Fernandes N., Jha V. (2024). Assisted peritoneal dialysis: Position paper for the ISPD. Perit. Dial. Int..

[9] Lim W.H., Dogra G.K., McDonald S.P., Brown F.G., Johnson D.W. (2011). Compared with younger peritoneal dialysis patients, elderly patients have similar peritoni-tis-free survival and lower risk of technique failure, but higher risk of peritonitis-related mortality. Perit. Dial. Int..

[10] Guo Q., Chen Y., Wu R., Yang L., Zhu X., Zhao Q., Zhuang X., Wu Y., Luo P., Cui W. (2022). Poorer clinical outcomes of early-onset peritonitis in elderly peritoneal dialysis patients: A longitu-dinal and multicenter study. Ther. Apher. Dial..

[11] Feng S., Wang Y., Qiu B., Wang Z., Jiang L., Zhan Z., Jiang S., Shen H. (2016). Impact of early-onset peritonitis on mortality and technique survival in peritoneal dialysis patients. SpringerPlus.

[12] Fourtounas C., Savidaki E., Dousdabanis P., Hardalias A., Kalliakmani P., Papachristou E., Drakopoulos A., Goumenos D.S., Vlachojannis J.G. (2006). Peritonitis during the first year after commencement of peritoneal dialysis has an impact on technique survival and patient morbidity. Adv. Perit. Dial..

[13] Harel Z., Wald R., Bell C., Bargman J.M. (2006). Outcome of patients who develop early-onset peritonitis. Adv. Perit. Dial..

[14] Hsieh Y.P., Wang S.C., Chang C.C., Wen Y.K., Chiu P.F., Yang Y. (2014). The negative impact of early peritonitis on continuous ambulatory peritoneal dialysis patients. Perit. Dial. Int..

[15] Martin L.C., Caramori J.C., Fernandes N., Divino-Filho J.C., Pecoits-Filho R., Barretti P. (2011). Geographic and educational factors and risk of the first peritonitis episode in Brazilian Peritoneal Dialysis study (BRAZPD) patients. Clin. J. Am. Soc. Nephrol..

[16] Pulliam J., Li N.C., Maddux F., Hakim R., Finkelstein F.O., Lacson E.J. (2014). R First-year outcomes of incident peritoneal dialysis patients in the United States. Am. J. Kidney Dis..

[17] Wang Z., Jiang L., Feng S., Yang L., Jiang S., Zhan Z., Song K., Shen H. (2015). Early peritonitis is an independent risk factor for mortality in elderly peritoneal dialysis patients. Kidney Blood Press Res..

[18] Radtke M., Albrektsen G.E., Wideroe T.E., Nilsen T.I., Romundstad P., Hallan S., Aasarod K., Laegreid I.K., Oien C. (2004). Changes in water transport across the peritoneum during treatment with continuous ambulatory peritoneal dialysis in selected patients with and without peritonitis. Perit. Dial. Int..

[19] van Diepen A.T., van Esch S., Struijk D.G., Krediet R.T. (2014). The first peritonitis episode alters the natural course of peritoneal membrane characteristics in peritoneal dialysis patients. Perit. Dial. Int..

[20] Chavers B.M., Solid C.A., Gilbertson D.T., Collins A.J. (2007). Infection-related hospitalization rates in pediatric versus adult patients with endstage renal disease in the United States. J. Am. Soc. Nephrol..

[21] Chow K.M., Li P.K.T., Cho Y., Abu-Alfa A., Bavanandan S., Brown E.A., Cullis B., Edwards D., Ethier I., Hurst H. (2023). ISPD catheter-related infection recommendations: 2023 update. Perit. Dial. Int..

[22] Pollmann L., Pollmann N.S., Jürgens C., Özcan F., Brinkhoff A., Schmeding M. (2025). Surgical risk factors for technical survival of peri-toneal dialysis catheters. Langenbecks Arch. Surg..

[23] Kwan J.R., Chong T.T., Low G.Z., Low G.W., Htay H., Foo M.W., Tan C. (2019). Outcomes following peritoneal dialysis catheter removal with reinsertion or permanent transfer to haemodialysis. J. Vasc. Access..

